# Exposure-Associated Physiologic Reactions (EAPR): A Novel Clinical Framework for Multisystem Adverse Responses

**DOI:** 10.7759/cureus.107645

**Published:** 2026-04-24

**Authors:** Akhtar Purvez, Ana Mir, Mudhasir Bashir

**Affiliations:** 1 Clinical Research, Momentum Medical Research, Charlottesville, USA; 2 Clinical Sciences, Lincoln Memorial University DeBusk College of Osteopathic Medicine, Harrogate, USA; 3 Clinical Sciences, Momentum Medical Research, Charlottesville, USA; 4 Psychiatry and Behavioral Sciences, University of Virginia, Charlottesville, USA

**Keywords:** adverse drug reactions, autonomic dysregulation, drug-induced arrhythmia, eapr, environmental exposures, exposure-associated physiologic reactions, mast cell activation, multisystem adverse reactions, non-ige hypersensitivity, precision medicine

## Abstract

Exposure-associated physiologic reactions (EAPR) represent unexpected physiologic responses to medications, food additives, environmental exposures, and immunologic stimuli that are increasingly encountered in modern clinical practice. While most exposures are well-tolerated, a subset of individuals develops disproportionate, multisystem responses that do not conform to traditional models of hypersensitivity, toxicity, or pharmacologic effect. These reactions are frequently misattributed to primary disease processes due to their atypical and transient nature, leading to diagnostic uncertainty and unnecessary investigation. Emerging clinical observations suggest that such responses may represent a broader and previously underrecognized phenomenon. This review proposes the concept of EAPR as a unifying clinical framework describing non-classical, often non-IgE-mediated responses to pharmacologic, dietary, environmental, and immunologic exposures. Drawing upon case-based evidence involving cardiovascular, renal, neurologic, ophthalmologic, and autonomic manifestations, this article integrates diverse observations into a cohesive mechanistic and diagnostic model. Recognition of EAPR may improve diagnostic accuracy, reduce unnecessary investigations, and enhance pharmacovigilance by integrating fragmented clinical observations into a coherent and clinically actionable framework. This framework is intended as a conceptual and hypothesis-generating model and requires further validation through systematic investigation.

## Introduction and background

Modern populations exist within an increasingly complex and dynamic exposure landscape characterized by continuous interaction with pharmacologic agents, industrially processed food additives, environmental chemicals, and immunologic stimuli. Advances in medicine, food technology, and biotechnology have significantly expanded both the diversity and frequency of these exposures, often in historically uncommon combinations. While the majority of individuals tolerate such exposures without clinically significant consequences, a subset develops disproportionate physiologic responses involving multiple organ systems [1]. The initial observations in this context have been prominently described in relation to food additives, but similar patterns have been reported across a broader range of pharmacologic, environmental, and immunologic exposures.

These reactions frequently present with acute or subacute symptom clusters that are temporally related to exposure yet do not conform to established diagnostic patterns. In routine clinical practice, such presentations are often misattributed to primary disease processes due to their atypical features and multisystem involvement. As a result, patients may undergo extensive diagnostic evaluations without identification of a unifying etiology [1,2].

Traditional frameworks for adverse reactions, centered on IgE-mediated hypersensitivity, predictable pharmacologic side effects, and dose-dependent toxicity, have provided a foundational structure for clinical reasoning. However, these models do not adequately capture the full spectrum of observed responses in contemporary clinical practice. Increasing recognition of non-IgE-mediated hypersensitivity, autonomic dysregulation, neuroinflammatory processes, and interindividual metabolic variability suggests that a broader conceptual approach is required to explain these phenomena [1,3-5]. Such responses may involve autonomic dysregulation, non-IgE-mediated mast cell activation, and interindividual metabolic or inflammatory variability. While established models, such as IgE-mediated hypersensitivity and dose-dependent toxicity, effectively explain predictable allergic reactions and pharmacologic side effects, they are less suited to account for multisystem, transient, and individually reproducible responses that lack clear immunologic markers or dose relationships. An exposure-associated physiologic reaction (EAPR) is intended to address this gap by providing a framework for recognizing these atypical, exposure-associated physiologic patterns.

A growing body of case-based literature, including multiple reports by Purvez and colleagues, demonstrates recurring patterns of temporally associated physiologic disturbances following exposure to otherwise widely tolerated agents. These include atrial fibrillation associated with dietary additives and pharmacologic therapies, ophthalmologic complications related to drug-induced physiologic changes, and neurologic syndromes following immune stimulation [2,6-11].

This framework is particularly relevant in the modern era of increasing exposure complexity, where traditional diagnostic models may fail to capture individualized physiologic responses. The recognition of EAPR, therefore, may present a useful evolution in clinical reasoning, bridging the gap between classical adverse reaction models and complex exposure-driven physiology [12,13].

## Review

Conceptual and clinical framework of EAPR

EAPR is proposed as a unifying clinical construct that describes a distinct category of physiologic responses occurring in susceptible individuals following exposure to pharmacologic agents, dietary components, environmental substances, or immunologic stimuli. EAPR refers to reproducible, exposure-linked physiologic responses in susceptible individuals that involve one or more organ systems and are not fully explained by established models of allergic, toxic, or pharmacologic reactions. Unlike traditional adverse reactions, which are typically predictable, dose-dependent, or mediated through well-defined immunologic pathways, EAPR suggests a broader and more complex interaction between external exposures and intrinsic host susceptibility.

EAPR is intended as a conceptual, hypothesis-generating clinical framework to aid in the recognition and organization of exposure-associated physiologic patterns, rather than a discrete, fully defined biologic syndrome with fixed mechanistic boundaries.

The need for such a framework arises from the growing recognition that many clinical presentations do not conform to established diagnostic paradigms. Patients frequently present with acute, multisystem symptoms that are temporally associated with specific exposures but lack the defining characteristics of classical allergic reactions, toxic effects, or primary disease processes. For example, patients may present with palpitations or atrial fibrillation following the ingestion of specific food additives, flushing and gastrointestinal symptoms related to sulfite exposure, or neurologic and ophthalmologic symptoms temporally associated with pharmacologic agents. These presentations are often transient, reproducible within individuals, and reversible upon withdrawal of the triggering exposure, suggesting an underlying physiologic mechanism rather than structural pathology [1,2].

It is important to recognize that underlying cardiac predisposition, including structural heart disease or baseline arrhythmic susceptibility, may confound the interpretation of these events. In clinical practice, differentiation relies on the reproducibility of episodes in relation to specific exposures, the absence of alternative triggers, and the resolution or reduction of events with targeted avoidance. These features may help distinguish exposure-associated physiology from background arrhythmic risk, although causality is not fully explained and definitively established in all cases.

EAPR is therefore best conceptualized as a systems-level phenomenon, integrating elements of autonomic regulation, immune activation, metabolic variability, and neurophysiologic signaling. Rather than acting through a single pathway, these reactions likely result from the convergence of multiple mechanisms, including non-IgE-mediated mast cell activation, autonomic nervous system imbalance, and individual differences in enzymatic and metabolic processing [3-5,13].

Future studies may help further characterize this subtype through objective markers of mast cell activation, such as serum tryptase, histamine metabolites, or other inflammatory mediators, although their clinical utility in this context remains to be established.

At the same time, the breadth of this systems-level framing may overlap with existing syndromic and mechanistic categories, including functional disorders, autonomic syndromes, and non-IgE-mediated hypersensitivity states. EAPR is not intended to replace or redefine these entities, but rather to provide a unifying clinical perspective that may help integrate and contextualize such presentations. Further refinement of boundaries will likely require additional empirical investigation. In this context, EAPR differs from existing categories by emphasizing the temporal relationship between specific exposures and reproducible multisystem responses, while remaining conceptually linked to these conditions through shared physiologic mechanisms.

This multidimensional nature explains both the variability of clinical presentations and the difficulty in categorizing these reactions within existing frameworks. A central principle of EAPR is individualized susceptibility. Evidence from case-based observations suggests that specific patients may exhibit consistent and reproducible responses to particular exposures, while the same exposures remain well tolerated in the broader population [2,6-11].

This aligns closely with emerging concepts in precision medicine, where genetic, metabolic, and immunologic variability play a critical role in determining physiologic responses [12,13]. In this context, EAPR may represent a clinically observable manifestation of underlying biologic heterogeneity.

Importantly, EAPR does not seek to replace existing models of adverse reactions but rather to complement and extend them. By providing a structured framework for recognizing atypical, multisystem, and exposure-related presentations, EAPR may allow clinicians to move beyond fragmented diagnostic approaches. This is particularly relevant in modern clinical environments, where patients are exposed to an increasing number of synthetic compounds, processed foods, and novel therapeutics.

From a practical standpoint, the EAPR framework may provide both diagnostic and therapeutic value. Recognition of exposure-related patterns may help reduce unnecessary diagnostic testing, minimize the misclassification of symptoms as primary disease, and guide targeted avoidance strategies. Furthermore, it provides a conceptual foundation for future research into biomarkers of susceptibility, mechanistic pathways, and individualized risk assessment [12-14].

Proposed classification of EAPR

To further refine the conceptual utility of EAPR, a preliminary classification system is proposed based on dominant physiologic pathways and clinical manifestations. While EAPR suggests a spectrum rather than discrete categories, this classification provides a practical framework for clinical recognition, research standardization, and hypothesis generation.

EAPR can be broadly categorized into four overlapping but clinically distinguishable subtypes: autonomic-dominant, immunologic/mast cell-dominant, electrophysiologic/cardiac, and metabolic/idiosyncratic. These categories are not mutually exclusive, and individual patients may exhibit features spanning multiple domains, reflecting the integrative nature of the underlying mechanisms. This broader categorization, which includes slower and more organ-specific manifestations, highlights the heterogeneity of EAPR and the need for more refined diagnostic criteria in future studies. Further work will be necessary to determine how such diverse presentations can be systematically classified within a unified framework.

Autonomic-dominant EAPR

This subtype is characterized by predominant involvement of the autonomic nervous system, with symptoms such as flushing, diaphoresis, tachycardia, palpitations, lightheadedness, and visceral discomfort. These reactions are often rapid in onset and transient, frequently resolving with removal of the triggering exposure. Mechanistically, they are thought to reflect dysregulation of the sympathetic-parasympathetic balance, potentially triggered by chemical or metabolic stimuli.

Autonomic-dominant EAPR is particularly relevant in cases involving food additives, such as sulfites or monosodium glutamate, where rapid-onset symptoms may mimic anxiety disorders, panic attacks, or gastrointestinal pathology. Recognition of this subtype is critical to avoid misdiagnosis and unnecessary interventions.

It is important to note that autonomic-dominant EAPR may overlap clinically with panic-spectrum and functional autonomic disorders. While formal exclusion of these conditions may not always be feasible, careful clinical evaluation is necessary to assess the temporal relationship to specific exposures and to distinguish reproducible exposure-linked patterns from primary functional or psychiatric conditions.

Immunologic/mast cell-dominant EAPR

This subtype encompasses reactions mediated by non-IgE-dependent mast cell activation or other immune pathways. Clinical features may include flushing, gastrointestinal discomfort, vasodilation, and systemic symptoms that resemble allergic reactions but lack classical markers such as urticaria or bronchospasm.

Unlike traditional hypersensitivity reactions, these responses may occur without prior sensitization and may be triggered by direct chemical stimulation of mast cells or secondary signaling pathways. Sulfite-related reactions may represent a prototypical example of this subtype.

Electrophysiologic/cardiac EAPR

This subtype is defined by cardiac manifestations, particularly arrhythmias such as atrial fibrillation, occurring in temporal association with specific exposures. Observations of recurrent atrial fibrillation linked to dietary additives and pharmacologic agents suggest that certain triggers may influence cardiac electrophysiology through autonomic modulation, ion channel effects, or metabolic perturbations.

The reproducibility of these events within individual patients supports a physiologic mechanism rather than coincidence. Recognition of this subtype has significant clinical implications, as it may prevent unnecessary long-term antiarrhythmic therapy when exposure avoidance is sufficient.

Metabolic/idiosyncratic EAPR

This subtype includes organ-specific reactions likely driven by metabolic variability, enzymatic differences, or idiosyncratic susceptibility. Clinical manifestations may include neurologic syndromes or ophthalmologic complications following exposure to medications or other agents. These reactions are often less immediately reversible than autonomic forms but still suggest a clear temporal relationship with exposure.

**Figure 1:**
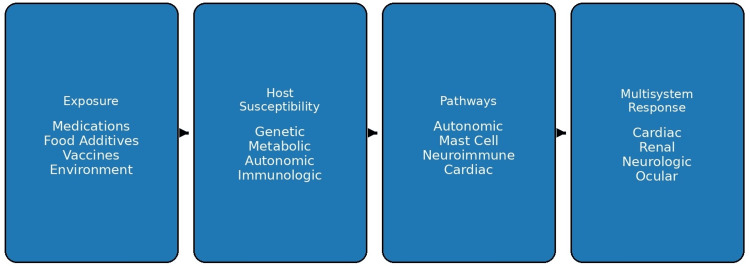
Conceptual model of exposure-associated physiologic reactions This figure illustrates the proposed conceptual framework of exposure-associated physiologic reactions (EAPR), suggesting the interaction between external exposures and intrinsic host susceptibility. Environmental, pharmacologic, dietary, and immunologic triggers act upon a susceptible individual, leading to activation of overlapping physiologic pathways, including autonomic dysregulation, non-IgE-mediated mast cell activation, neuroinflammatory processes, and metabolic variability. These converging mechanisms result in multisystem clinical manifestations affecting cardiovascular, neurologic, gastrointestinal, renal, and ophthalmologic systems. The model emphasizes the nonlinear, systems-level nature of EAPR, highlighting the transition from exposure to integrated physiologic response rather than isolated organ-specific pathology. Image Credits: Akhtar Purvez, Ana Mir, Mudhasir Bashir. Information synthesized from referenced literature [1,4-6,13].

The overall conceptual framework of EAPR, integrating exposure triggers, host susceptibility, and downstream multisystem responses, is illustrated in Figure 1.

The defining characteristics, underlying mechanisms, clinical spectrum, and diagnostic approach associated with EAPR are summarized in the following tables.

The defining features of EAPR are summarized in Table 1.

**Table 1:** Defining characteristics of exposure-associated physiologic reactions Table Credits: Akhtar Purvez, Ana Mir, Mudhasir Bashir. Information synthesized from referenced literature [1,2,4-6].

Feature	Description	Clinical Relevance
Exposure-linked onset	Temporal relationship	Establish causality
Multisystem involvement	Multiple organs	Suggest systemic response
Non-classical mechanisms	Non-IgE-mediated	Avoid misclassification
Reproducibility	Recurs in individuals	Strengthens diagnosis
Reversibility	Improves with withdrawal	Confirms trigger
Diagnostic ambiguity	Frequently misdiagnosed	Avoid unnecessary testing

**Figure 2:**
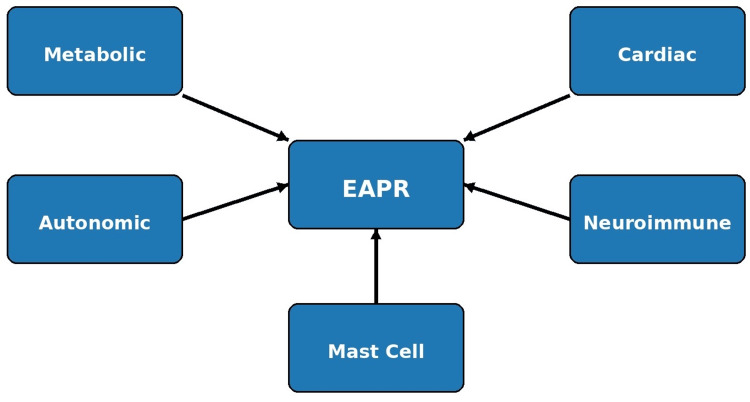
Integrated pathophysiologic pathways in exposure-associated physiologic reactions (EAPR) This diagram depicts the interconnected mechanisms underlying EAPR, emphasizing the central role of autonomic dysregulation, non-IgE-mediated mast cell activation, neuroinflammation, metabolic variability, and cardiac electrophysiologic modulation. These pathways are shown converging to produce heterogeneous but reproducible physiologic responses in susceptible individuals, highlighting the systems-level nature of the EAPR framework. Image Credits: Akhtar Purvez, Ana Mir, Mudhasir Bashir. Information synthesized from referenced literature [2-8,13].

The principal overlapping pathophysiologic pathways proposed in EAPR are summarized in Table 2 and schematically illustrated in Figure 2.

**Table 2:** Pathophysiologic mechanisms of exposure-associated physiologic reactions (EAPR) Table Credits: Akhtar Purvez, Ana Mir, Mudhasir Bashir. Information synthesized from referenced literature [2-8,13-15].

Mechanism	Description	Clinical Manifestations	Evidence
Autonomic dysregulation	ANS imbalance	Flushing, tachycardia	[5,6]
Mast cell activation	Non-IgE mediated	GI, vasodilation	[4]
Neuroinflammation	Immune-mediated	Neurologic syndromes	[3]
Cardiac electrophysiology	Ion channel/autonomic	Atrial fibrillation	[2,7,8]
Metabolic variability	Enzymatic/genetic	Susceptibility	[13-15]

Representative exposure-response patterns described in the current literature are outlined in Table 3.

**Table 3:** Clinical spectrum of exposure-associated physiologic reactions (EAPR) Table Credits: Akhtar Purvez, Ana Mir, Mudhasir Bashir. Information synthesized from referenced literature [2,7-12]. Note: The examples listed may represent a range of clinical observations with varying levels of evidentiary support. Some associations are supported by recurrent case-based observations, while others are more preliminary and hypothesis-generating. Rechallenge should only be considered when clinically safe and ethically appropriate, particularly in cases where prior reactions have been significant.

Exposure	Category	Reaction
MSG	Food additive	Atrial fibrillation
Sulfites	Preservative	Autonomic syndrome
Tirzepatide	Medication	Atrial fibrillation
Iron sucrose	Medication	Acute kidney injury
Duloxetine	Medication	Acute kidney injury
Topiramate	Medication	Angle-closure glaucoma

**Figure 3:**
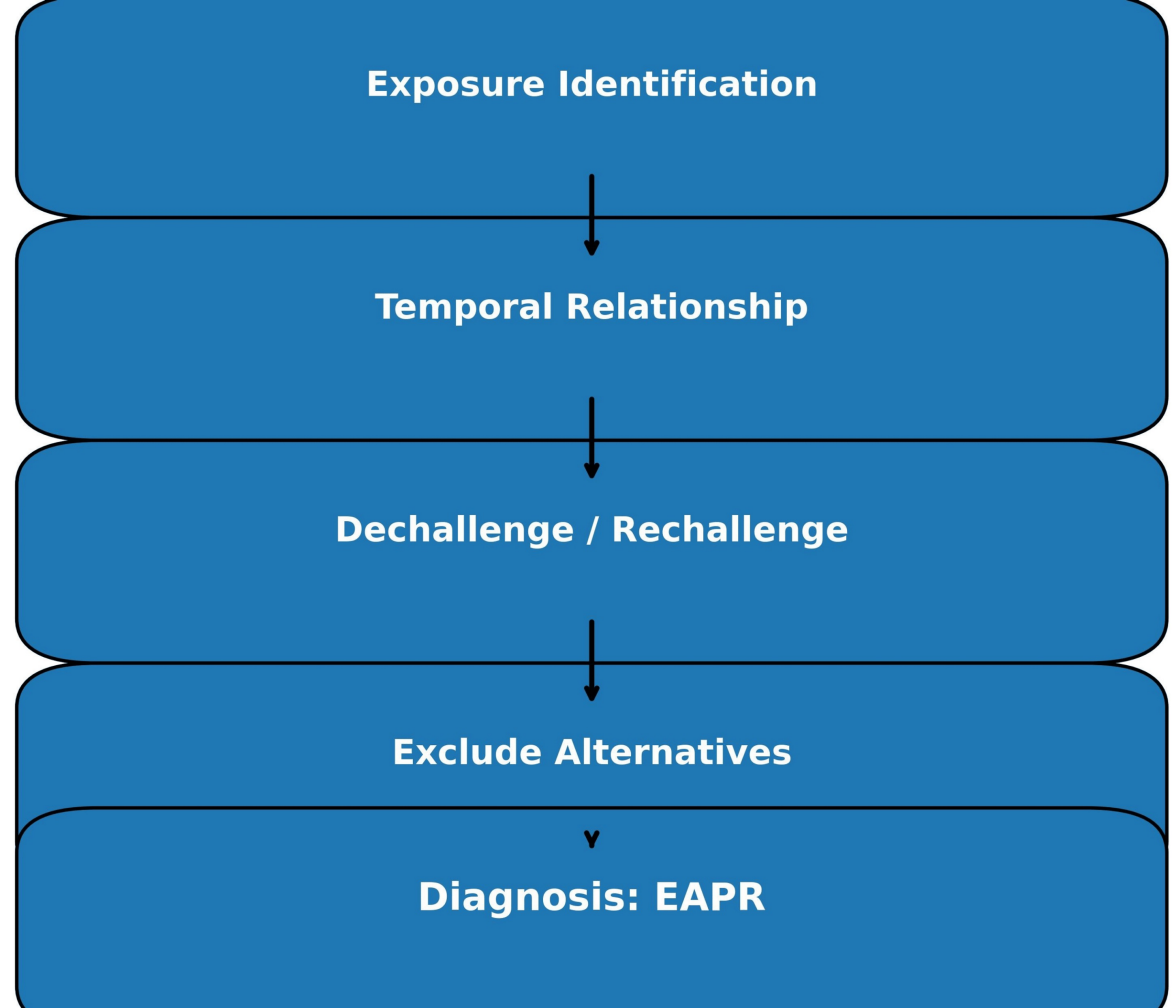
Diagnostic approach to exposure-associated physiologic reactions (EAPR) This flow diagram outlines a structured clinical approach to identifying EAPR, including an assessment of exposure history, evaluation of temporal relationships, response to dechallenge, consideration of rechallenge when appropriate, and exclusion of alternative diagnoses. The algorithm emphasizes pattern recognition and exposure linkage as central components of diagnosis. Image Credits: Akhtar Purvez, Ana Mir, Mudhasir Bashir. Information synthesized from referenced literature [1,2,6,13].

A practical clinical approach to recognizing EAPR is shown in Table 4. A structured clinical approach to the identification and diagnosis of EAPR is outlined in Figure 3.

**Table 4:** Diagnostic framework for exposure-associated physiologic reactions (EAPR) Table Credits: Akhtar Purvez, Ana Mir, Mudhasir Bashir. Information synthesized from referenced literature [1,2,6].

Step	Clinical Action	Purpose
Exposure history	Review triggers	Identify cause
Temporal relationship	Timing analysis	Establish link
Dechallenge	Withdrawal	Confirm
Rechallenge	Re-exposure	Strengthen
Exclusion	Rule out others	Avoid misdiagnosis

Discussion

The concept of EAPR may represent a useful extension of existing clinical frameworks, emerging in response to a growing body of observations that are not fully explained by traditional models of adverse reactions [1-3]. It is important to emphasize that the current framework is primarily derived from case-based observations and clinical pattern recognition, and therefore should be interpreted as exploratory and hypothesis-generating rather than definitively validated.

A defining feature of EAPR is individualized host susceptibility. The reproducibility of reactions within individual patients suggests these events reflect consistent physiologic responses rather than random occurrences. This aligns with precision medicine concepts, where genetic, metabolic, and immunologic variability influences responses to environmental exposures [12,13].

Multiple reports by Purvez and colleagues further reinforce the EAPR framework, demonstrating consistent patterns across diverse clinical domains, including monosodium glutamate-associated atrial fibrillation, tirzepatide-associated arrhythmia, and topiramate-induced angle-closure glaucoma [2,6-11].

The autonomic nervous system appears to play a central role in EAPR. Clinical manifestations, such as flushing, diaphoresis, tachycardia, and visceral discomfort, suggest dysregulation of sympathetic and parasympathetic balance. These symptoms are often rapid in onset and reversible, indicating a functional rather than structural mechanism [5,6]. While autonomic involvement may be a common organizing feature across many presentations, it is not necessarily universal, and other mechanisms may predominate in specific subtypes.

Another unifying mechanism is non-IgE-mediated mast cell activation. Unlike classical allergic responses, these reactions may occur through direct chemical stimulation of mast cells or alternative signaling pathways, leading to mediator release without traditional allergic markers [3]. Direct mechanistic confirmation for many of these pathways remains limited in the current evidence base, and these proposed mechanisms should be interpreted as biologically plausible but not yet definitively established.

Cardiac electrophysiologic effects are particularly significant. Observations of atrial fibrillation associated with dietary additives and medications suggest that autonomic imbalance, ion channel modulation, and metabolic perturbations may converge to alter myocardial excitability [2,7,8].

EAPR also encompasses neurologic and ophthalmologic manifestations, further supporting its multisystem nature. Neurologic syndromes and ophthalmologic complications following pharmacologic exposure highlight the diversity of organ involvement [9,11].

From a systems biology perspective, EAPR may be conceptualized as an interface phenomenon in which external exposures interact with intrinsic host susceptibility to produce nonlinear physiologic responses. This framework aligns with broader models of personalized and network-based medicine [13].

While several illustrative cases in this manuscript are derived from a single author group, similar patterns of individualized, exposure-associated physiologic responses have also been described in independent literature, particularly in relation to food additives, autonomic symptoms, and non-IgE-mediated reactions. These external observations, considered alongside the authors’ case-based reports, support the broader applicability of the proposed framework while also underscoring its still exploratory and hypothesis-generating nature. Similar patterns have also been described across broader literature involving food additive sensitivity, mast cell-mediated responses, and autonomic or inflammatory pathways, supporting the generalizability of these observations [16-28].

Clinically, EAPR is frequently misdiagnosed due to its atypical presentation. Patients often undergo extensive investigations before recognition of an exposure-related cause. Early identification allows for targeted avoidance strategies and improved outcomes.

The framework also has important implications for pharmacovigilance. Case reports serve as critical early signals of previously unrecognized adverse effects and contribute to pattern recognition across populations [6].

In routine clinical practice, reproducibility is often inferred through consistent temporal associations between exposure and symptom onset, resolution following avoidance, and recurrence with unintentional re-exposure. Formal rechallenge may not be feasible or ethically appropriate in many cases, particularly when prior reactions have been severe. In such settings, careful longitudinal observation and patient-reported exposure patterns play a critical role in establishing clinical plausibility.

Despite its strengths, the EAPR framework has limitations. Much of the supporting evidence is derived from case reports, which lack statistical power and control groups. However, the consistency of clinical patterns, temporal associations, and biologic plausibility supports the plausibility of the framework.

Future research should focus on identifying biomarkers of susceptibility, exploring genetic and metabolic predictors, and conducting prospective studies.

In summary, EAPR may represent a useful unifying clinical framework that integrates diverse observations into a coherent model of individualized physiologic reactivity.

## Conclusions

Exposure-associated physiologic reactions (EAPR) are proposed as an underrecognized yet clinically significant clinical framework characterized by individualized, multisystem responses to commonly encountered pharmacologic, dietary, environmental, and immunologic exposures. These reactions may reflect a broader and reproducible pattern of physiologic reactivity that is insufficiently explained by traditional models of adverse effects.

Recognition of EAPR may require a shift toward exposure-centered clinical reasoning, incorporating careful exposure history and awareness of atypical presentations. Early identification may improve diagnostic accuracy, reduce unnecessary investigations, and enable targeted prevention strategies, supporting a more individualized approach to patient care. This framework may serve as a foundation for future mechanistic studies and prospective validation in precision medicine. Further validation through systematic and prospective studies will be necessary to establish its clinical applicability and generalizability.

## References

[1] Vally H, Misso NL, Madan V (2009). Clinical effects of sulphite additives. Clin Exp Allergy.

[2] Purvez A, Valentine CM, Monfredi OJ, Engel GD (2023). Recurrent atrial fibrillation episodes related to monosodium glutamate (MSG): a case report. Anaesth Pain Intensive Care.

[3] Akin C (2017). Mast cell activation syndromes. J Allergy Clin Immunol.

[4] Joyner MJ, Paneth N (2015). Seven questions for personalized medicine. JAMA.

[5] Vandenbroucke JP (1999). Case reports in an evidence-based world. J R Soc Med.

[6] Goldstein DS, Robertson D, Esler M, Straus SE, Eisenhofer G (2002). Dysautonomias: clinical disorders of the autonomic nervous system. Ann Intern Med.

[7] Parsons A, Johnstone A (2001). Postcode prescribing and the Human Rights Act 1998. J R Soc Med.

[8] Benarroch EE (2020). Physiology and pathophysiology of the autonomic nervous system. Continuum (Minneap Minn).

[9] Benarroch EE (2007). The autonomic nervous system. Basic anatomy and physiology. Continuum (Minneap Minn).

[10] Schaumburg HH, Byck R, Gerstl R, Mashman JH (1969). Monosodium L-glutamate: its pharmacology and role in the Chinese restaurant syndrome. Science.

[11] Purvez A, Valentine CM, Bashir M (2025). Monosodium glutamate and atrial fibrillation in a 78-year-old woman: a case report. Ann Intern Med Clin Cases.

[12] Howell C, Wilson AD, Waring WS (2007). Cardiovascular toxicity due to venlafaxine poisoning in adults: a review of 235 consecutive cases. Br J Clin Pharmacol.

[13] Purvez A, Mirza M, Bashir M (2025). New-onset atrial fibrillation potentially associated with tirzepatide: a case report. Cureus.

[14] Purvez A, Bao NT, Bashir M (2025). Topiramate-induced angle-closure glaucoma in a healthy female: a case report. Anaesth Pain Intensive Care.

[15] Lester MR (1995). Sulfite sensitivity: significance in human health. J Am Coll Nutr.

[16] Collins FS, Varmus H (2015). A new initiative on precision medicine. N Engl J Med.

[17] Tracey KJ (2002). The inflammatory reflex. Nature.

[18] Young E, Patel S, Stoneham M, Rona R, Wilkinson JD (1987). The prevalence of reaction to food additives in a survey population. J R Coll Physicians Lond.

[19] Theoharides TC, Valent P, Akin C (2015). Mast cells, mastocytosis, and related disorders. N Engl J Med.

[20] Steindler DA (2023). Sugar substitutes and taste enhancers need more science, sensitivity- and allergy-guided labeling. NPJ Sci Food.

[21] Simon RA (2003). Adverse reactions to food additives. Curr Allergy Asthma Rep.

[22] McCann D, Barrett A, Cooper A (2007). Food additives and hyperactive behaviour in 3-year-old and 8/9-year-old children. Lancet.

[23] Stevenson DD, Simon RA (1981). Sensitivity to ingested metabisulfites in asthmatic subjects. J Allergy Clin Immunol.

[24] Yang WH, Purchase EC (1985). Adverse reactions to sulfites. CMAJ.

[25] Vally H, Thompson PJ (2001). Role of sulfite additives in wine induced asthma: single dose and cumulative dose studies. Thorax.

[26] Raithel M, Baenkler HW, Naegel A (2005). Significance of salicylate intolerance in diseases of the lower gastrointestinal tract. J Physiol Pharmacol.

[27] Allen DH, Delohery J, Baker G (1987). Monosodium glutamate-induced asthma. J Allergy Clin Immunol.

[28] Purvez A, Mir A, Bashir M (2026). Multisystem immune, autonomic, thromboembolic and vestibular dysfunction after mRNA COVID-19 vaccination: a case report. Ann Intern Med Clin Cases.

